# Sleep Quality Prediction From Wearable Data Using Deep Learning

**DOI:** 10.2196/mhealth.6562

**Published:** 2016-11-04

**Authors:** Aarti Sathyanarayana, Shafiq Joty, Luis Fernandez-Luque, Ferda Ofli, Jaideep Srivastava, Ahmed Elmagarmid, Teresa Arora, Shahrad Taheri

**Affiliations:** ^1^ Qatar Computing Research Institute Hamad Bin Khalifa University Qatar Foundation Doha Qatar; ^2^ Department of Medicine Weill Cornell Medical College in Qatar, Qatar Foundation Doha Qatar

**Keywords:** wearables, sleep quality, sleep efficiency, actigraphy, body sensor networks, mobile health, connected health, accelerometer, physical activity, pervasive health, consumer health informatics, deep learning

## Abstract

**Background:**

The importance of sleep is paramount to health. Insufficient sleep can reduce physical, emotional, and mental well-being and can lead to a multitude of health complications among people with chronic conditions. Physical activity and sleep are highly interrelated health behaviors. Our physical activity during the day (ie, awake time) influences our quality of sleep, and vice versa. The current popularity of wearables for tracking physical activity and sleep, including actigraphy devices, can foster the development of new advanced data analytics. This can help to develop new electronic health (eHealth) applications and provide more insights into sleep science.

**Objective:**

The objective of this study was to evaluate the feasibility of predicting sleep quality (ie, poor or adequate sleep efficiency) given the physical activity wearable data during awake time. In this study, we focused on predicting good or poor sleep efficiency as an indicator of sleep quality.

**Methods:**

Actigraphy sensors are wearable medical devices used to study sleep and physical activity patterns. The dataset used in our experiments contained the complete actigraphy data from a subset of 92 adolescents over 1 full week. Physical activity data during awake time was used to create predictive models for sleep quality, in particular, poor or good sleep efficiency. The physical activity data from sleep time was used for the evaluation. We compared the predictive performance of traditional logistic regression with more advanced deep learning methods: multilayer perceptron (MLP), convolutional neural network (CNN), simple Elman-type recurrent neural network (RNN), long short-term memory (LSTM-RNN), and a time-batched version of LSTM-RNN (TB-LSTM).

**Results:**

Deep learning models were able to predict the quality of sleep (ie, poor or good sleep efficiency) based on wearable data from awake periods. More specifically, the deep learning methods performed better than traditional linear regression. CNN had the highest specificity and sensitivity, and an overall area under the receiver operating characteristic (ROC) curve (AUC) of 0.9449, which was 46% better as compared with traditional linear regression (0.6463).

**Conclusions:**

Deep learning methods can predict the quality of sleep based on actigraphy data from awake periods. These predictive models can be an important tool for sleep research and to improve eHealth solutions for sleep.

## Introduction

### Background

The importance of sleep is paramount to health and performance. Insufficient sleep can impede physical, emotional, and mental well-being [1,2] and can lead to a multitude of health complications such as insulin resistance [3-5], high blood pressure [6], cardiovascular disease [7,8], a compromised immune or metabolic system [9,10], mood disorders (such as depression or anxiety) [11,12], and decreased cognitive function for memory and judgment [13-15].

There are many indicators of sleep quality, an important one being sleep efficiency. Sleep efficiency is a metric that takes into consideration the time spent asleep, the time it takes to fall asleep, and the time asleep but with disturbance (ie, awakenings). Poor sleep efficiency can lead to sleep deprivation, which is found to be a major health risk with links to diseases such as diabetes and obesity [16]. Also, sleep behavior has been found to impact adolescent health [17-19]. Recent systematic reviews have shown the relevance of physical activity to sleep, including sleep efficiency [20-22]. Although the relationship between physical activity and sleep is not yet fully understood, it is thought to be a strong and complex correlation contributing to multiple lifestyle diseases such as type 2 diabetes mellitus and obesity [20-22]. However, the underlying mechanism between physical activity and sleep is not yet fully understood.

### Role of Wearables in Sleep Health and eHealth

There are many research tools to study the link between physical activity and sleep, including standardized questionnaires, actigraphy sensors, and polysomnography (PSG). All of these methods have different clinical indications (eg, diagnosis of different sleep disorders) [23]. For example, PSG is considered the “gold standard” for sleep medicine, as it involves the use of multiple sensors during sleep such as electroencephalogram, motion sensors, breathing sensors, SpO_2_(oxygen saturation), and so forth. These sensors monitor and observe a patient overnight [24] and can be used for diagnosing different sleep disorders. The elaborate nature of PSG means that it is generally limited to one overnight observation. Even the portable solutions, which permit PSG assessment in the patient’s home, are complex to perform and not without limitations [25,26].

To better understand the impact of daily physical activity on sleep behavior, new tools are needed. Sleep researchers in the early 1990s developed a technique called *actigraphy* to study sleep interactions using wearable devices [27]. Although actigraphy traditionally uses wearable devices to evaluate the sleep period of a patient, it can also be used to observe physical activity. Actigraphy has become a widely used tool, as it has been found to be much more reliable than subjective or self-reported sleep diaries and behavior logs [28]. Patients wear the device for a period of time as they continue their daily routines. The technique has been especially influential for large cohort studies where PSG is not feasible [29]. Moreover, actigraphy allows for the continuous longitudinal monitoring of a patient. This is particularly impactful for the study of diseases such as chronic obstructive pulmonary disease where sleep disturbances can be a predictor of exacerbation of the disease [30]. Current approaches to the analysis of actigraphy data involve sleep experts performing a number of steps manually. This is a bottleneck, and hence there may be troves of actigraphy data left unanalyzed.

Furthermore, there are hundreds of consumer-grade physical activity and sleep tracking devices (eg, Fitbit) collecting motion data similarly to actigraphy devices. These consumer devices are being used by millions of people collecting huge amounts of data. Some devices even allow the collection of data for very long periods (eg, several months), as they require only an occasional need for battery recharge (ie, Garmin VivoFit). Recent studies have found that these consumer-grade devices can sometimes have similar precision to clinical-grade actigraphy sensors [31]. There are successful examples of the integration of physical activity wearable data into eHealth tailored applications [32], including smart-watch health applications [33] that can collect physical activity and sleep data directly from the watch.

### Objective

As previously noted, physical activity and sleep are interrelated. In this study, we tested the feasibility of predicting poor or good sleep efficiency based on physical activity data from the awake periods from a wearable device (ie, actigraphy). We took a detour from classical methods and proposed deep learning approaches to modeling the relationship between sleep and physical activity. The importance of this research is two-fold.

First, since our approach can be used in cases where sleep sensor data is not available, our models can be used in the early detection of potential low sleep efficiency. This is a common problem with consumer-grade wearable devices, as users might not wear them during the night (battery recharging, sensors embedded in smart jewelry, and so forth).

Second, our study was focused on advanced deep learning methods. Traditional prediction models applied to activity raw accelerometer data (eg, logistic regression) suffer from at least 2 key limitations: (1) They are not robust enough to learn useful patterns from noisy raw accelerometer output. As a result, existing methods for classification and analysis of physical activity rely on extracting higher-level features that can be fed into prediction models [34]. This process often requires domain expertise and can be time consuming. (2) Traditional methods do not exploit task labels for feature construction, and thus can be limited in their ability to learn task-specific features. Deep learning has the advantage that it is robust to raw noisy data, and can learn, automatically, higher level abstract features by passing raw input signals through nonlinear hidden layers while also optimizing on the target prediction tasks. We leveraged this characteristic by building models using a range of deep learning methods on raw accelerometer data. This reduced the need for data preprocessing and feature space construction and simplified the overall workflow for clinical practice and sleep researchers.

## Methods

### Overview

The methodology of this study involved several steps: (1) the collection of wearable data with regards to physical activity and sleep patterns, (2) data processing and representation, (3) data modeling, and (4) performance evaluation. The following subsections explain each of these steps.

### Data Collection

The deidentified data used in this study were collected by Weill Cornell Medical College-Qatar for a research study called Qatar’s Ultimate Education for Sleep in Teenagers. The aim of the study was to determine different sleep patterns in adolescents residing in Qatar and how these related to body weight status. The institutional review board (IRB) approval was initially obtained by the joint IRB for Hamad Medical Corporation and Weill Cornell Medical College. The selected cohort was chosen from adolescents attending 1 of the 2 high schools and registered in grades 7-11. The dataset used contained complete actigraphy data from a subset of 92 adolescents over 1 week. There were 322 total sleep instances: 102 from boys and 220 from girls.

**Figure 1:**
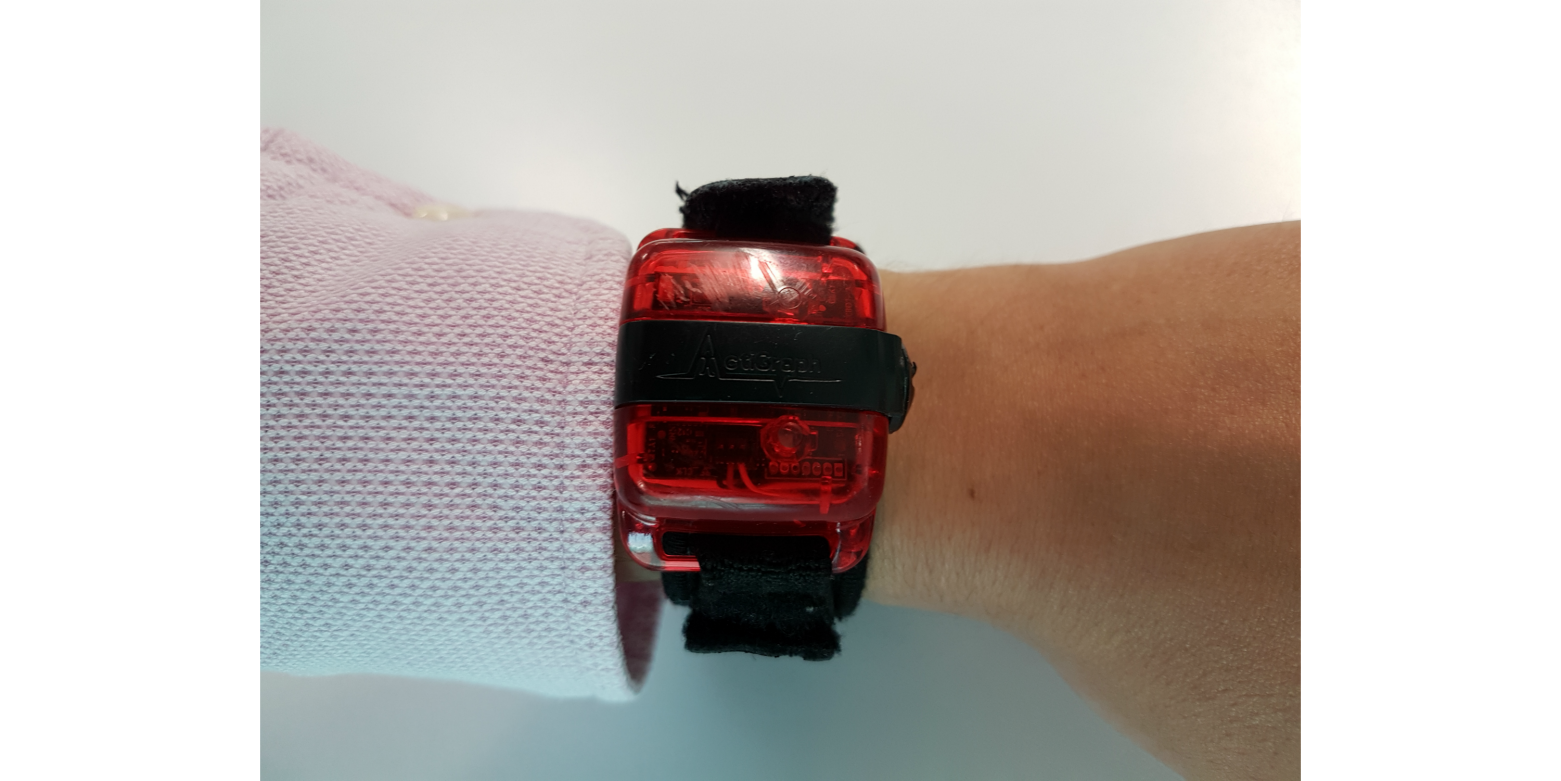
ActiGraph Gt3X+.

After agreeing to participate in the study, the adolescent participants were provided with an ActiGraph GT3X+ device, placed on their nondominant wrist, and were instructed to wear it at all times for 7 consecutive days and nights. The ActiGraph GT3X+, shown in Figure 1, is a clinical-grade wearable device that samples a user’s activity at 30-100 Hz (we used 30 Hz in this study). The effectiveness of this device has been successfully validated against clinical polysomnography [35]. The participants were instructed not to remove the water-resistant device at any time. We used the manufacturer’s software (ActiLife version 6; available from ActiGraph, LLC) to export the data. Although the device is triaxial, our methods used the vertical axis only.

The subjective self-reported sleep diaries were collected, but they were not considered for the study, since actigraphy data provides a more objective measurement of physical activity and sleep patterns. Sadeh’s and American Academy of Sleep Medicine sleep definitions were used in this interpretation [27,36,37]. For calculating the sleep efficiency score, we considered each individual sleeping period as sleep.

### Data Processing and Representation

#### Sleep Quality Definitions

In a person’s activity time series, that is, the continuous data collected from a wearable device, there are moments when an individual is awake and when they are asleep. The latter is referred to as the sleep period. The boundary from awake time to the sleep period is called the sleep onset time, and the boundary from sleep period to awake time is referred to as the sleep awakening time. The period of time between the self-reported time to bed and the sleep onset time is called the latency.

**Figure 2:**
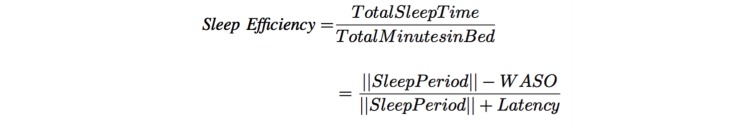
Sleep efficiency equation as defined by sleep specialists.

To measure sleep quality we determined sleep efficiency (see Figure 2), which is the ratio of total minutes asleep to total minutes in bed. Those achieving a sleep efficiency score of ≥85% are thought to be good-quality sleepers, and those with a score of <85% are thought to have poor-quality sleep [38].

**Figure 3:**
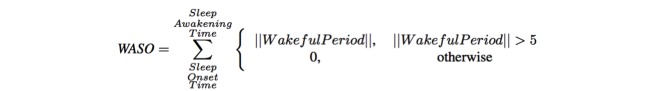
The adapted wake after sleep onset calculation.

Total minutes in bed represents the amount of time that an individual spends asleep as well as the amount of time the individual takes to fall asleep, that is, latency. Total sleep time represents the amount of time that an individual spends asleep, less the amount of time the person awakens. This is calculated by subtracting the wake after sleep onset (WASO) from the duration of the sleep period. WASO is the sum of all moments of wakefulness lasting longer than 5 minutes (see Figure 3).

#### Data Processing

The data collected from the actigraphy device contained triaxial accelerometer movement. Previous research has identified that for devices worn on the wrist, the vertical axis from accelerometer data is the most indicative of physical activity [39]. Consequently, we switched to 1-dimensional input for a simpler and less noisy modeling (ie, each row in our dataset contained a 1-dimensional time series of vertical axis accelerometer data).

**Figure 4:**
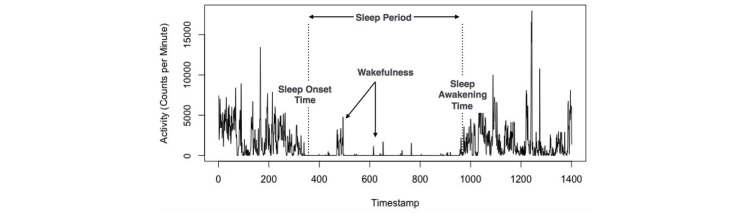
Example of sleep definitions on accelerometer data of an actigraphy device.

The raw accelerometer data was aggregated into minute-by-minute epochs using a script written in R version 3.3.1, a statistical Open Source computing software developed as part of the R Project. In this dataset, the time series contains activity data during both the *awake time* and the *sleep time* (as shown in Figure 4). We used the awake time to form the prediction (ie, as the model input) and the sleep time to determine the ground truth of sleep quality. Sleep quality (good or poor) was determined from the sleep time actigraphy data, and each time series was labeled as such.

As mentioned earlier, sleep onset time and sleep awakening time are metrics that form the boundary of the sleep period [36]. We interpreted and expanded these values for accelerometer data according to Sadeh’s actigraphy definitions [37].

**Figure 5:**
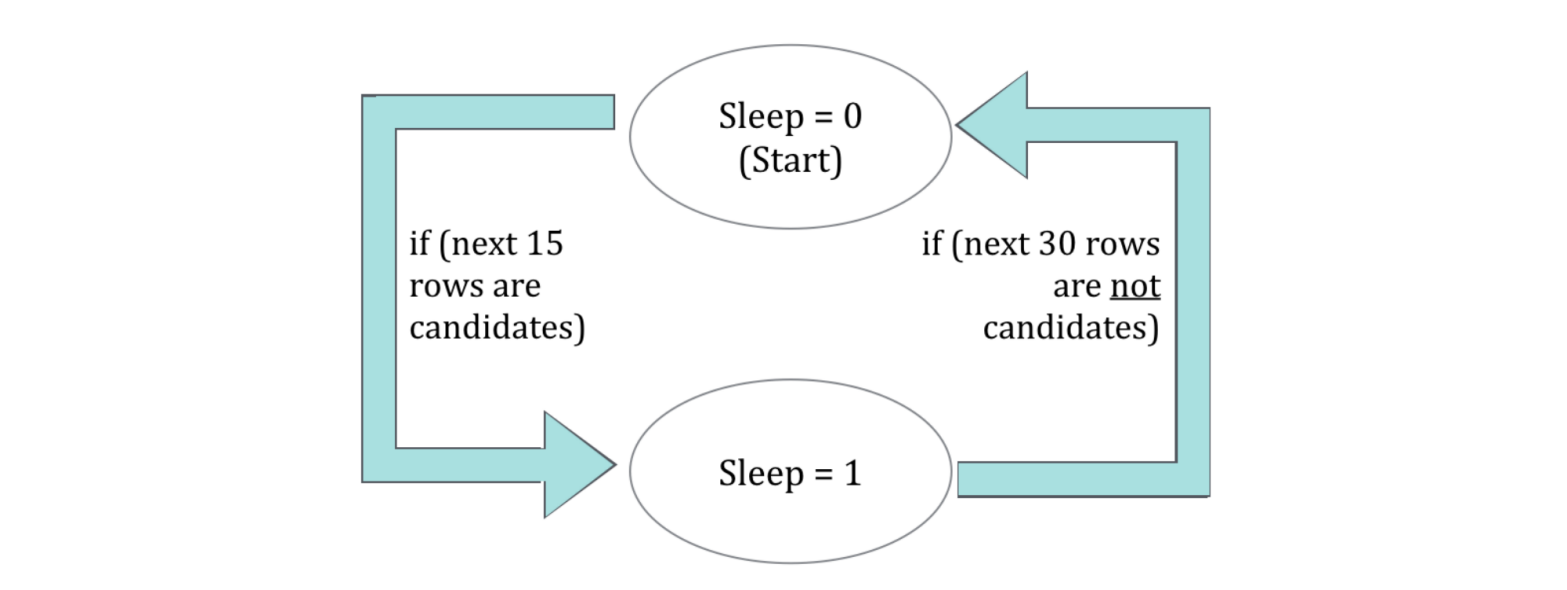
State machine diagram explaining the designation of sleep or awake time periods.

*Sleep onset time* is traditionally defined as the first minute of 15 continuous minutes of sleep after a self-reported bedtime, and the *sleep awakening time* is the last minute of 15 continuous minutes of sleep that is followed by 30 minutes of movement [37]. To automate this interpretation directly from the accelerometer output, we developed the concept of candidate rows. *Candidate rows* denote moments (or designated epochs) in time with a lack of triaxial movement and require a subsequent pass to determine whether the individual is asleep or awake. Each row is iterated upon and run through a state machine, as illustrated in Figure 5. Since there is no self-reported bedtime, we inferred it as the beginning of sedentary behavior immediately preceding and adjacent to the start of the sleep period. The duration of this sedentary time is the *latency*.

The handling of nonwearing time is very important and should be minimized wherever possible. The device was water-resistant and did not require recharging during the time period the participants were instructed to wear it. If a participant removed the device, the triaxial accelerometer would record zero values for the time it was not worn. Natural human behavior makes micro-movements that are sensed by the accelerometer even during sleep, and so periods with a continuous lack of movement indicate device removal. In other words, during the time the device was not worn, our algorithm would identify candidate rows for the entire period, denote them as sleep time, and score the sleep as having a perfect sleep efficiency of 1. Alternatively, the ActiLife software includes an algorithm to remove nonwear periods from physical activity calculations [40].

### Models for Sleep Quality

As explained in more detail below, in this study we explored the use of deep learning methods to predict sleep quality based on actigraphy data. We compared the results of our deep learning models to those of logistic regression, a standard statistical method. In this section, we explain how each model was built. The models used in our study were as follows:

- Logistic regression, a nondeep learning model
- Multi-layer perceptrons (MLPs), a deep learning model
- Convolutional neural network (CNN), a deep learning model
- Recurrent neural networks (RNN), a deep learning model
- Long short-term memory (LSTM) RNN, a deep learning model
- Time-batched long short-term memory (TB-LSTM) RNN, a deep learning model

For logistic regression, we fed the input signals directly into the output layer for prediction. In contrast, for the deep learning models, we passed the inputs through one or more hidden layers before they were fed to the output layer for prediction. Each hidden unit in the hidden layers used a nonlinear activation function. In our study, we experimented with different hidden layer units using rectified linear unit as our activation function.

#### Data Partitioning

To train our models without over-fitting and test their performance afterward, we created a random partitioning of the dataset. Each time series was assigned to a partition randomly while maintaining an even class distribution of the target variable, sleep quality. The data were split with a 70%-15%-15% ratio for training, testing, and validation sets, respectively.

#### Input and Output of the Models

The input of the models were time series vectors, *X=(x*_1_*, · · · , x*_T_*)*, representing the physical activity of a person’s awake time. Each vector corresponded to a continuous period of awake time, and so for each individual, there might be multiple such vectors over the 7 days. Each *x*_T_ represented the value of the vertical axis at time *t*.

The output of the model was a binary classification decision between *good* and *poor* sleep quality based on sleep efficiency (%). These classifications corresponded to the sleep efficiency definitions as described earlier. In addition to the binary decision, the model also gave its confidence (a score between 0.0 and 1.0) in that decision.

#### Training the Models

To be able to predict, we first trained the models on the training dataset. We used an online training algorithm RMSprop [41], which relied on a number of preset parameters:

- Mini-batch size: how many training instances to consider at one time.
- Learning rate: the rate at which parameters are updated.
- Max epoch: maximum number of iterations over the training set.
- Dropout ratio: ratio of hidden units to turn off in each mini-batch training.

The training algorithm minimizes the cross-entropy between the predicted distribution and the actual (gold) target labels. To avoid over-fitting, we used early stopping based on the model’s performance on the validation set. In particular, we evaluated the model after every epoch on the validation set and stopped when its accuracy went down. To reduce the cross-entropy between the predicted distributions and the target distributions, RMSprop was used setting the maximum number of epochs to 50 as suggested by the authors [41].

#### Logistic Regression (a Nondeep Learning Model)

As a baseline, we used logistic regression (LR) to predict the sleep quality. LR is a generalized linear classification model that does not have any hidden layers. For the LR, the raw input signals *X* are directly fed to the output layer for prediction without any nonlinear hidden layer transformations. The optimal setting for logistic regression (LR) was with a mini-batch size of 5 and a dropout ratio of 0.5.

#### Multilayer Perceptrons (a Deep Learning Model)

MLPs, also known as feed-forward neural networks, are the simplest models in the deep learning family. They have one or more hidden layers. In fact, MLP without any hidden layers is equivalent to logistic regression. In MLP, all the units of a hidden layer are fully connected to the units in the previous layer. The best parameter configuration for MLP was with a mini-batch size of 20, a dropout ratio of 0.1, and a hidden layer size of 15.

#### Convolutional Neural Network (a Deep Learning Model)

CNNs are a more complex type of deep learning method that includes repetitive filters or kernels applied to local time slots, thereby composing a high level of abstract features. This operation is called *convolution*. After convolution, a *max-pooling* operation is performed to select the most significant abstract features. This design of CNNs yields fewer parameters than its fully connected counterpart (MLP), and therefore generalizes well for target prediction tasks. For its best configuration, we used 25 hidden nodes, filter length of 5 and pooling length of 4, 5 mini-batch size, and 0.0 dropout ratio.

#### Recurrent Neural Networks (a Deep Learning Models)

RNNs compose abstract features by processing activity measures in an awake time sequentially, at each time step combining the current input with the previous hidden state. RNNs create internal states by remembering the previous hidden layer, which allows them to exhibit dynamic temporal behavior. These features make RNNs a good deep learning method for temporal series. RNNs performed best with a mini-batch size of 5, a dropout ratio of 0.1 and a hidden layer size of 75. To avoid over-fitting, we used a technique based on dropout of hidden units and early stopping based on the loss on the development set [42].

#### Long Short-Term Memory (a Deep Learning Model)

A subtype of RNN, *LSTM* uses specifically designed memory blocks as units in the recurrent layer to capture longer-range dependencies. The optimal configuration values for LSTM were a mini batch size of 5, dropout ratio of 0.5, and hidden layer size of 100.

#### Time-Batched Long Short-Term Memory (a Deep Learning Model)

To further improve our implementation of LSTM, we constructed batches of time steps by merging accelerometer measures over time steps. We referred to this version of the model as *TB-LSTM*. The configuration values for TB-LSTM were mini-batch size of 5, dropout ratio of 0.5, and hidden layer size of 100.

### Performance Evaluation

For the evaluation of the performance of the different models, we reported on several well-known metrics such as accuracy, precision, recall, F1-score, and area under the receiver operating characteristic (ROC) curve (AUC). These metrics are commonly used in data mining and clinical decision support systems.

#### Accuracy

It is computed as the proportion of correct predictions, both positive and negative (sum of true positives and true negatives divided by the number of all instances in the dataset).

#### Precision

It is the fraction of the number of true positive predictions to the number of all positive predictions (ie, true positives divided by the sum of true positives and false positives). In our case, precision described what percentage of the time the model predicted “good-quality sleep” correctly. Note that precision is also known as positive predictive value.

#### Specificity

It is the fraction of the number of true negative predictions to the actual number of negative instances in the dataset (ie, true negatives divided by the sum of true negatives and false positives). In our case, specificity referred to the percentage of the correctly predicted “poor-quality sleep” to the total number of “poor-quality sleep” instances in the dataset. Note that specificity is also known as true negative rate.

#### Recall or Sensitivity

It is the fraction of the number of true positive predictions to the actual number of positive instances in the dataset (ie, true positives divided by the sum of true positives and false negatives). In our case, recall referred to the percentage of the correctly predicted “good-quality sleep” to the total number of “good-quality sleep” instances in the dataset. Note that recall is also known as true positive rate or sensitivity.

#### F1-Score

There is usually an inverse relationship between precision and recall. That is, it is possible to increase the precision at the cost of decreasing the recall, or vice versa. Therefore, it is more useful to combine them into a single measure such as F1 score, which computes the harmonic mean of precision and recall.

#### Area Under the ROC Curve

It represents the probability that a classifier will rank a randomly chosen positive instance higher than a randomly chosen negative instance. Hence, AUC defines an effective and combined measure of sensitivity and specificity (which are often inversely related, just like precision and recall) for assessing inherent validity of a classifier.

## Results

### Comparison Between Deep Learning and Linear Regression

**Figure 6:**
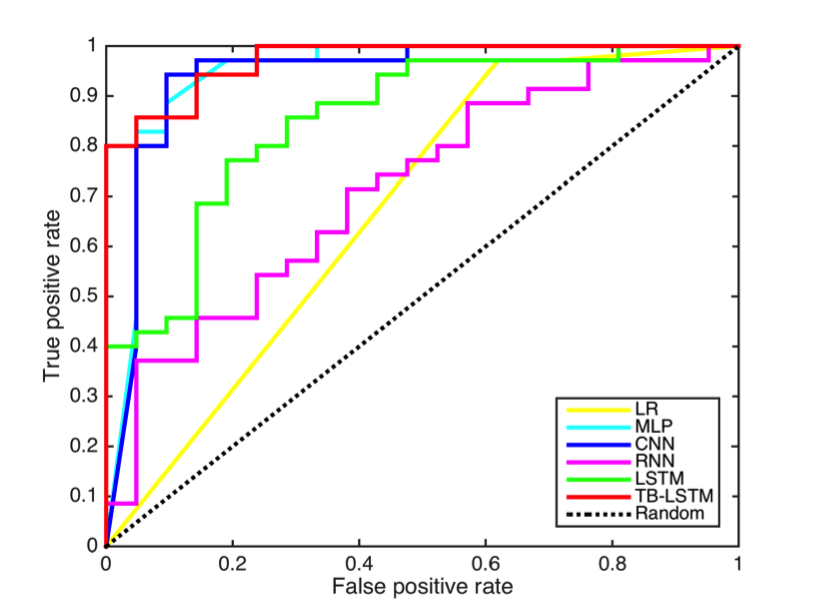
Receiver operating characteristic (ROC) curves for each model’s prediction of sleep efficiency.

As shown in Table 1 and Figure 6, the performance of the linear regression in the metrics previously explained performed worse than the models based on deep learning. Only the simple RNN performed worse than linear regression in both F1-score (harmonic mean of precision and recall) and accuracy.

**Table 1:** Results on raw accelerometer data.

Data models	AUC^a^	F1-Score	Precision	Recall	Accuracy
LR^b^	0.6463	0.8193	0.7083	0.9714	0.7321
MLP^c^	0.9449	0.9118	0.9394	0.8857	0.8929
CNN^d^	0.9456	0.9444	0.9189	0.9714	0.9286
RNN^e^	0.7143	0.7711	0.6667	0.9143	0.6607
LSTM^f^-RNN	0.8531	0.8500	0.7556	0.9714	0.7857
TB-LSTM^g^	0.9714	0.9211	0.8537	1.000	0.8929

^a^AUC: Area under receiver operating characteristic (ROC) curve.

^b^LR: logistic regression.

^c^MLP: Multilayer perceptrons.

^d^CNN: convolutional neural networks.

^e^RNN: recurrent neural networks.

^f^LSTM: long short-term memory.

^g^TB-LSTM: time-batched LSTM.

As shown in Table 1, the AUC of the linear regression model was low. The AUC value for LR was 0.6463, which was close to 0.5 (equivalent to a random prediction). This showed the limitation of classical models in analyzing raw accelerometry.

In contrast, all the AUC values for the deep learning models were better with a range from 0.7143 to 0.9714, TB-LSTM being the best performer and RNN, the worst. Time-batched LSTM, CNN, and MLP performed the best with AUC scores showing an improvement over LR by 50%, 46%, and 46%, respectively.

### Comparison Between Deep Learning Models

Upon comparing the deep learning neural network models, we noticed that CNN yielded slight improvement over MLP in AUC (0.07% absolute), but more in F1 (3.57%) and accuracy (4.00%). These improvements could be attributed to the time- invariant convolution-pooling operations of the CNN model to pick key local patterns, which generalized well for small training data. The F1-score improved by 12% for time-batched LSTM, by 15% for CNN, and by 11% for MLP. The accuracy improved by 22% for time-batched LSTM, by 27% for CNN, and by 22% for MLP.

A comparison of the RNN models revealed that LSTM outperformed simple RNN by a wide margin; 19%, 10%, and 19% in AUC, F1, and accuracy, respectively. These gains over simple RNNs could be attributed to the specially designed gates of LSTMs that could capture long-range dependencies between physical activities in the sequences.

However, this is not surprising. Both simple and LSTM RNNs operate on sequences, where each time step comprises only one activity value. This often results in very long sequences. As mentioned earlier, in this setting RNNs cannot compose higher-level features effectively because of low-dimensional input at each time step, and also suffer from vanishing gradient problems due to lengthy sequences.

Our solution to surmount this problem was to use a time-batched input to LSTM. When we compared the results of our time-batched LSTM (TB-LSTM) with those of MLP and CNN, we found that TB-LSTM outperformed both MLP and CNN in AUC by 3%; in fact, it had the highest AUC score. It achieved better F1 score than MLP (1%), but worse than CNN (–2%). When we observed their precision and recall values, we found that TB-LSTM had a very high recall but lower precision, which meant that it tended to predict more goods than gold standard. For the same reason, its accuracy was also lower than that of CNN.

## Discussion

### Principal Findings

In our study, we focused on the prediction of poor versus good sleep efficiency. That is a simple, but important, problem, as sleep efficiency has been found to be a crucial sleep parameter with important health consequences [38,43,44]. Furthermore, we did not quantify in our prediction the overall sleep efficiency but simply the differentiation between two classes (poor versus good sleep efficiency). This classification is consequently not an indicator of sleep patterns, but the prediction of a sleep quality parameter that might indicate a potential sleep problem.

As in prediction or diagnostic problems, our results need to be discussed in terms of sensitivity and specificity (see Table 2). The deep learning methods of CNN and TB-LSTM were the best performers overall. Their sensitivity (0.97 and 1, respectively) showed that these models were able to detect nearly all the cases of “good-quality sleep,” meaning that in a tool for screening potential sleep problems these models will be able to detect easily people with normal sleep quality. Often high sensitivity comes at the price of low specificity (ie, failing to identify negative cases, or true negative error). This was the case of linear regression, which had a high sensitivity but a specificity of 0.3, meaning that in such models many “poor sleeps” would have been wrongly classified as good sleep. This is very important, since misidentifying poor sleep cases can lead to underdiagnosis of problematic sleep.

**Table 2:** Sensitivity and specificity results.

Data models	Sensitivity or recall	Specificity
LR^a^	0.9714	0.333
MLP^b^	0.8857	0.9048
CNN^c^	0.9714	0.8571
RNN^d^	0.9143	0.2381
LSTM^e^	0.9714	0.4762
TB-LSTM^f^	1.000	0.7143

^a^LR: logistic regression.

^b^MLP: multilayer perceptrons.

^c^CNN: convolutional neural networks.

^d^RNN: recurrent neural networks.

^e^LSTM: long short-term memory.

^f^TB-LSTM: time-batched LSTM.

The sensitivity (also known as recall) and specificity of each of the models are reported in Table 2. The high sensitivity values of each of the models indicate that deep learning has a strong capability of correctly identifying individuals with good sleep patterns from their preceding awake activity. The specificity is high for TB-LSTM, MLP, and CNN, indicating that these models were also able to successfully distinguish those with poor sleep patterns.

### Relevance of Findings

Previous algorithms, such as by Sadeh et al [27,37], do not focus on the prediction of sleep quality based on physical activity during awake time, but rather on prediction of sleep quality based on accelerometer data during the sleep periods. The objective of those studies was the validation of the actigraphy data compared with PSG. Consequently, the objectives and findings of those studies cannot be compared with our study.

There are several data analytics areas relevant to our work. To our knowledge, our research was the first one looking into the use of deep learning for the study of actigraphy data related to physical activity and sleep. Previous research on the use of deep learning for sleep science has been focused on PSG data [45,46]. In other application areas, deep learning has been used for human activity recognition [47,48] which is a similar technical problem. In a previous study, we combined human recognition of actigraphy data with other machine learning algorithms, but not deep learning [49].

#### Impact in Sleep Science

Sleep insufficiency is highly prevalent in contemporary society, and has been shown to influence energy balance by altering metabolic hormone regulation. Consequently, health researchers are exploring the impact of sleep and physical activity on many health conditions. A major bottleneck for this research is that current approaches for studying actigraphy data require intensive manual work by human experts. Furthermore, huge datasets of actigraphy data are emerging from health research, including the study of sleep disorder patients, healthy populations, and epidemiological studies. Furthermore, millions of consumers are buying wearables that incorporate activity sensors. This burst of human activity data is a great opportunity for health research, but to achieve this paradigm shift, it is necessary to develop new algorithms and tools to analyze this type of data.

As explained in the results, our findings supported the feasibility of using physical activity data to predict the quality of sleep in terms of sleep efficiency. These findings were by no means aiming to substitute well-studied algorithms and methods for studying sleep and physical activity data, such as the methods described by Sadeh [37]. Improved algorithms, such as the ones we presented in this study, for actigraphy analysis can lead to a paradigm shift in the study of lifestyle behaviors such as sleep and physical activity, just as electrocardiography became crucial for cardiology and clinical research.

Our research showed that deep learning performed better than classical methods in terms of learning useful patterns from raw accelerometer data for the task of sleep quality prediction. Since deep learning models compute abstract features from raw input signals while optimizing on the actual sleep quality, this process yields a more robust solution. Furthermore, the good results of deep learning showed that raw accelerometer data had more “signal” regarding sleep quality, which traditional models such as linear regression are not able to capture right now. More research needs to be done to understand why deep learning performs better, which eventually can help in identifying new factors influencing the quality of sleep.

#### Impact in eHealth

Our study provided an early example of how advanced deep learning methods could be used to infer new insights from raw actigraphy data. Our focus on predicting and forecasting can help design new eHealth applications where predictions are made to personalize coaching for patients or to facilitate decision making of professionals.

There is an increased interest in sleep in the health domain. This is consequently being reflected in an increase use of eHealth for sleep [50-54] and also in the use of social media for sharing sleep logs [53]. The expansion of eHealth into sleep is not limited to sleep disorders, but also to improve sleep for people living with chronic conditions such as cancer [53]. These developments are closely related to the concept of Quantified Self for health [55,56]. Furthermore, we can assume that predicting sleep quality based on physical activity data acquired by accelerometer data (both from actigraphy or activity trackers) can be used to provide personalized feedback, such as momentary ecological interventions based on mobile technology [57].

In our study, we attempted to predict a parameter regarding the quality of sleep solely relying on the physical activity during the awake time. To our knowledge, this was not done earlier. The advantage of this approach is that eventually the same approach can be used to predict sleep quality with data from smart watches and other wearable devices that are not necessarily used during sleep. Therefore, our models can eventually be used within eHealth applications that do not require wearing a sensor during sleep. This is of special interest for the development of smart watch health apps [33], as they might require frequent battery charging.

Our research has many limitations as explained in the next subsection. However, this is the first study, to our knowledge, that focused on the prediction of sleep quality from physical activity accelerometer data during awake periods. Our methodology and results can be used as the baseline for further studies looking into predicting sleep quality from mobile and wearable devices. This is a source of major concern, since many sleep apps in the making predict with unclear methodology and performance [50-52]. Although more studies are highlighting the increasing reliability of consumer sleep wearables [31], we do not know how they calculate or predict sleep quality parameters. To maximize the potential of the wearable mass adoption for sleep health, we need research on not only the reliability of consumer-grade devices but also their data processing and modeling techniques.

### Limitations

There were some limitations in our study regarding the generalization of our results. Sleep behavior can be affected by cultural aspects and also change with age. Our study sample drew sleep data from adolescents, aged 10-17 years, living in Qatar. Future research will need to evaluate whether applications of deep learning for sleep research using actigraphy will yield similar results in different populations (eg, adults and people with chronic conditions).

In our study, the prediction was simplified to “good” and “poor” sleep quality with regards to sleep efficiency. This may be an oversimplification of complex sleep problems. To provide more precise predictions (eg, quantitative value of sleep quality), these techniques will need to be validated. A prediction, such as the one presented in this study, might be useful for the detection of people with unhealthy sleep patterns, but not to identify the causes of poor sleep efficiency.

There is also a limitation in the interpretation of deep learning. Deep learning models are “black boxes” and do not provide explanation of their sleep efficiency prediction. Other techniques such as linear regression can provide insights on which features contribute to the prediction. However, this study showed that the performance of such models was much lower than that of deep learning. New techniques in deep learning are being researched to facilitate the interpretation of such models.

In our study, the models used the data from the individual’s awake time to predict of sleep quality. The prediction was made at the last moment before sleep using the full awake time activity. If these models were to be used to provide personalized feedback to individuals with sleep problems, they will need to be tested with fragments of the awake time, giving an individual time to alter their behavior. Since our data was fragmented into sleep periods and awake times specific to an individual, the models would be able to handle varying durations of awake time.

### Conclusions

Our study showed the feasibility of deep learning in predicting sleep efficiency using wearable data from awake periods. This is of paramount importance because deep learning eliminated the need for data preprocessing and simplified the overall workflow in sleep data research. The feasibility of our approach can lead to new applications in sleep science and also to the development of more complex eHealth sleep applications for both professionals and patients. These models can also be integrated in the broader context of quantified self [55,56].

## Abbreviations

AUC: Area under ROC curve
CNN: convolutional neural network
LR: logistic regression
LSTM-RNN: long short-term memory RNN
MLP: multilayer perceptron
PSG: polysomnography
ROC: receiver operating characteristic
RNN: recurrent neural network
SpO2: oxygen saturation
TB-LSTM: time-batched version of LSTM-RNN
