# Correction of: Sleep Quality Prediction From Wearable Data Using Deep Learning

**DOI:** 10.2196/mhealth.6953

**Published:** 2016-11-25

**Authors:** Aarti Sathyanarayana, Shafiq Joty, Luis Fernandez-Luque, Ferda Ofli, Jaideep Srivastava, Ahmed Elmagarmid, Teresa Arora, Shahrad Taheri

**Affiliations:** ^1^Qatar Computing Research InstituteHamad Bin Khalifa UniversityQatar FoundationDohaQatar; ^2^Department of MedicineWeill Cornell Medical College in Qatar, Qatar FoundationDohaQatar

The authors of “Sleep Quality Prediction From Wearable Data Using Deep Learning” (JMIR Mhealth Uhealth 2016;4(4):e125) have mistakenly written “linear regression” instead of “logistic regression” in the following 9 instances. Linear regression cannot be used for binary classification, and was not utilized in this methodology. In all the tables and in most of the cases authors wrote correctly “logistic regression,” however this typo needs to be fixed to avoid confusion in the following 9 instances:

 1. In the results section within the abstract, the sentence “More specifically, the deep learning methods performed better than traditional linear regression” should be changed to “More specifically, the deep learning methods performed better than traditional logistic regression.”

2. In the results section within the abstract, the sentence “CNN had the highest specificity and sensitivity, and an overall area under the receiver operating characteristic (ROC) curve (AUC) of 0.9449, which was 46% better as compared with traditional linear regression (0.6463).” should be changed to “CNN had the highest specificity and sensitivity, and an overall area under the receiver operating characteristic (ROC) curve (AUC) of 0.9449, which was 46% better as compared with traditional logistic regression (0.6463).”

3. The first subheading of the results section in the body of the paper, “Comparison Between Deep Learning and Linear Regression” should be changed to “Comparison Between Deep Learning and Logistic Regression.”

4. Under the first subheading in the results section of the text, the first sentence, “As shown in Table 1 and Figure 6, the performance of the linear regression in the metrics previously explained performed worse than the models based on deep learning” should be changed to “As shown in Table 1 and Figure 6, the performance of the logistic regression in the metrics previously explained performed worse than the models based on deep learning.”

5. Under the first subheading in the results section of the text, the second sentence, “Only the simple RNN performed worse than linear regression in both F1-score (harmonic mean of precision and recall) and accuracy” should be changed to “Only the simple RNN performed worse than logistic regression in both F1-score (harmonic mean of precision and recall) and accuracy.”

6. Under the first subheading in the results section of the text, the third sentence, “As shown in Table 1, the AUC of the linear regression model was low. The AUC value for LR was 0.6463, which was close to 0.5 (equivalent to a random prediction)” should be changed to “As shown in Table 1, the AUC of the logistic regression model was low. The AUC value for LR was 0.6463, which was close to 0.5 (equivalent to a random prediction).”

7. Under the subheading Prinicpal Findings in the discussion section, “This was the case of linear regression, which had a high sensitivity but a specificity of 0.3, meaning that in such models many ‘poor sleeps’ would have been wrongly classified as good sleep” should be changed to “This was the case of logistic regression, which had a high sensitivity but a specificity of 0.3, meaning that in such models many ‘poor sleeps’ would have been wrongly classified as good sleep.”

8. Under the subheading of relevance of findings in the discussion section, “Furthermore, the good results of deep learning showed that raw accelerometer data had more ‘signal’ regarding sleep quality, which traditional models such as linear regression are not able to capture right now” should be changed to “Furthermore, the good results of deep learning showed that raw accelerometer data had more ‘signal’ regarding sleep quality, which traditional models such as logistic regression are not able to capture right now.”

9. Under the subheading Limitations in the discussion section, “Other techniques such as linear regression can provide insights on which features contribute to the prediction” should be changed to “Other techniques such as logistic regression can provide insights on which features contribute to the prediction.”

All these alterations have been made in the online version of the paper on the JMIR website on November 25, 2016, together with publishing this correction notice. Because these were made after submission to PubMed and other full-text repositories, the correction notice has been submitted to PubMed, and the original paper has been resubmitted to PubMed Central. The corrected metadata have also been updated on PubMed manually and were resubmitted to CrossRef.

